# Central Pontine Myelinolysis and Hypokalemic Paralysis as Initial Manifestations of Primary Sjögren’s Syndrome: A Rare Electrolyte-Mediated Neurological Presentation

**DOI:** 10.7759/cureus.106980

**Published:** 2026-04-13

**Authors:** Rohith Srinivas, Preethi GK

**Affiliations:** 1 Internal Medicine, ESIC Medical College and Post Graduate Institute of Medical Science and Research (PGIMSR), Bangalore, IND; 2 Neurology, St. John's Medical Academy, Bangalore, IND

**Keywords:** autoimmune disease, central pontine myelinolysis, distal renal tubular acidosis, hypokalemia, sjogrens syndrome

## Abstract

A 63-year-old woman presented with acute quadriparesis and severe hypokalemia (2.07 meq/L) secondary to distal renal tubular acidosis (dRTA), which was later attributed to primary Sjögren’s syndrome (SS). Brain MRI revealed a characteristic trident-shaped hyperintense lesion in the central pons on T2-weighted and fluid-attenuated inversion recovery (FLAIR) sequences, consistent with central pontine myelinolysis (CPM). The patient had no history of rapid sodium correction, suggesting hypokalemia as the primary trigger for CPM. She was treated with intravenous potassium replacement and intravenous methylprednisolone, followed by oral steroids and hydroxychloroquine. Her neurological symptoms resolved completely within one month, and follow-up MRI showed only residual gliosis. This case highlights two rare neurological manifestations of SS - hypokalemic paralysis and CPM - both resulting from electrolyte disturbances rather than direct autoimmune-mediated nerve damage. While peripheral neuropathy is the most common neurological complication of SS, central nervous system involvement, particularly CPM, is exceedingly rare. This report emphasizes the importance of considering SS in patients with unexplained hypokalemia and acute neurological deficits, as timely diagnosis and treatment can lead to favorable outcomes.

## Introduction

Primary Sjögren's syndrome (SS) is a chronic autoimmune disorder primarily characterized by lymphocytic infiltration of exocrine glands, leading to the classic sicca complex of xerostomia and keratoconjunctivitis sicca. However, approximately 30-50% of patients develop systemic manifestations, including renal, pulmonary, and neurological involvement [1,2].

Central nervous system (CNS) involvement in primary SS is uncommon. While earlier studies reported highly variable rates (2-25% of neurological cases), more recent large cohort data suggest a lower frequency, with CNS manifestations occurring in approximately 3-5% of patients. Peripheral neuropathy remains the most common neurological complication [3-6].

Renal involvement in primary SS most commonly presents as distal renal tubular acidosis (dRTA), which can lead to severe hypokalemia and, rarely, hypokalemic paralysis. Severe electrolyte disturbances may also precipitate osmotic demyelination syndromes such as central pontine myelinolysis (CPM), although this association is rarely described in SS.

dRTA in primary SS results from autoimmune-mediated destruction of intercalated cells in the collecting duct, with dysfunction of H⁺-ATPase pumps. This leads to impaired urinary acidification, non-anion gap metabolic acidosis, and secondary renal potassium wasting. When severe, hypokalemia can cause muscle weakness or acute flaccid paralysis.

We present an unusual case of primary SS manifesting with acute quadriparesis secondary to hypokalemic paralysis due to dRTA, with concomitant CPM, highlighting the complex interplay between electrolyte disturbances and neurological injury in autoimmune disease.

## Case presentation

A 63-year-old woman presented with a seven-day history of progressive fatigue and generalized weakness, culminating in acute-onset flaccid quadriparesis over the preceding 24 hours. She reported mild xerostomia for two months but denied other sicca symptoms.

On examination, her blood pressure was 110/70 mmHg and pulse rate was 88 beats/minute, regular in rhythm. Higher mental functions were normal, although mild dysarthria was noted. Neurological examination revealed severe symmetric weakness (Medical Research Council (MRC) grade 2/5 in the upper limbs and 1/5 in the lower limbs) with generalized hyporeflexia. There were no sensory deficits, and cranial nerve examination was normal.

Laboratory investigations as in Table 1 demonstrated severe hypokalemia (2.07 mEq/L; normal 3.5-5.0 mEq/L) with renal potassium wasting (urinary potassium 21.99 mEq/L). Arterial blood gas analysis revealed non-anion gap metabolic acidosis (HCO₃⁻ 7.6 mEq/L, pCO₂ 18 mmHg). Urine anion gap was positive (+18 mEq/L) , supporting impaired ammonium excretion consistent with dRTA. Creatine phosphokinase (CPK) was elevated (709.1 IU/L), suggesting myopathy. The elevated CPK level (709.1 IU/L) was attributed to hypokalemia-induced myopathy, as it normalized to 82 IU/L within 72 hours of potassium repletion. There were no clinical features suggestive of inflammatory myopathy, such as myalgias, muscle tenderness, or disproportionate proximal weakness. Electromyography and nerve conduction studies were not performed.

**Table 1:** Laboratory Investigations at Presentation

Parameter	Patient Value	Reference Range
Hemoglobin	13.2 g/dL	12–16 g/dL
Total Leukocyte Count	7.8 ×10³/µL	4–11 ×10³/µL
Platelet Count	260 ×10³/µL	150–450 ×10³/µL
Blood Urea	28 mg/dL	15–40 mg/dL
Serum Creatinine	0.9 mg/dL	0.6–1.3 mg/dL
Serum Sodium	141 mmol/L	135–145 mmol/L
Serum Potassium	2.07 mmol/L	3.5–5.0 mmol/L
Serum Chloride	104 mmol/L	98–106 mmol/L
Serum Calcium	7.38 mg/dL	8.5–10.5 mg/dL
Creatine Phosphokinase (CPK)	709.1 IU/L	26–192 IU/L
Urinary Potassium	21.99 mEq/day	15–40 mEq/day
Plasma Renin Activity	92.75 mIU/L	4.40–46.10 mIU/L
Aldosterone	13.30 ng/dL	2.21–35.30 ng/dL
Thyroid Stimulating Hormone (TSH)	0.608 µIU/mL	0.55–4.78 µIU/mL
Free T3	1.94 pg/mL	2.3–4.2 pg/mL
Free T4	1.43 ng/dL	1.0–1.6 ng/dL
Rheumatoid Factor	Negative	Negative
Antinuclear antibodies (ANA)	1:640 (Speckled)	Negative
Anti-SSA (Ro)	90 (+++) Positive	Negative
Anti-Ro52	103 (+++) Positive	Negative
Anti-SSB (La)	42 (++) Positive	Negative
Anti-nRNP/Sm	27 (++) Positive	Negative
Anti-Sm	Negative	Negative
Anti-Scl-70	Negative	Negative
Anti-PM-Scl100	Negative	Negative
Anti-Jo-1	Negative	Negative
Anti-Centromere B	Negative	Negative
Anti-dsDNA	Negative	Negative
Arterial pH	7.244	7.35–7.45
PaO₂	89.8 mmHg	80–100 mmHg
PaCO₂	17.9 mmHg	35–45 mmHg
Standard HCO₃⁻	17.2 mmol/L	22–26 mmol/L
Actual HCO₃⁻	19.7 mmol/L	22–26 mmol/L
Oxygen Saturation	94.2 %	95–100 %

Autoimmune evaluation showed positive antinuclear antibodies (ANA) (1:640, speckled pattern), strongly positive anti-Ro/SSA (>8.0 AI), and anti-La/SSB (6.2 AI). Plasma renin was elevated (92.75 mIU/L) with normal aldosterone levels (13.1 ng/dL), supporting a diagnosis of dRTA associated with SS. The diagnosis of primary SS was further supported by fulfilment of the 2016 American College of Rheumatology (ACR) and the European League Against Rheumatism (EULAR) classification criteria. Schirmer’s test was positive (3 mm/5 min; 1 point) and, along with strongly positive anti-Ro/SSA antibodies (3 points), fulfilled the 2016 ACR/EULAR classification criteria for primary SS (total score: 4). The low free T3 level (1.94 pg/mL; reference range 2.3-4.2 pg/mL), in the presence of normal thyroid stimulating hormone (TSH) and free T4 levels, was attributed to euthyroid sick syndrome in the setting of acute illness. Thyroid autoimmunity was not further evaluated.

**Figure 1:**
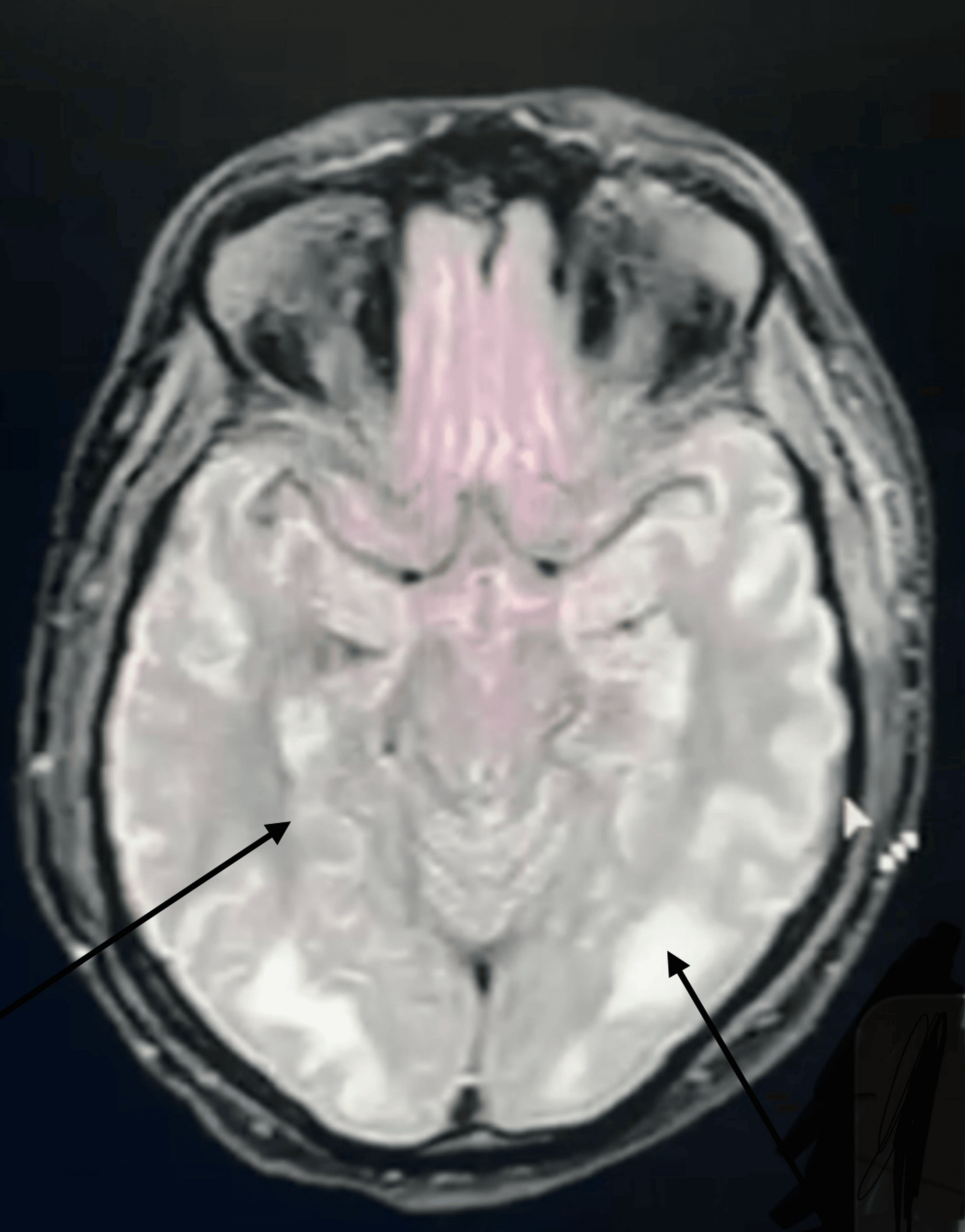
Axial T2-weighted brain MRI obtained on day three of hospitalization (10 days after symptom onset) showing a trident-shaped hyperintensity in the central pontine region, consistent with central pontine myelinolysis (CPM). Diffuse white matter hyperintensities suggest associated demyelination. No gadolinium contrast was administered

Magnetic resonance imaging of the brain demonstrated a trident-shaped, non-enhancing T2/fluid-attenuated inversion recovery (FLAIR) hyperintensity in the central pons, sparing the ventrolateral fibers and corticospinal tracts, characteristic of CPM as shown in Figure 1.

Notably, serum sodium levels remained normal (141 mmol/L) throughout hospitalization. The patient received aggressive potassium repletion with intravenous potassium chloride (40 mEq every 12 hours), followed by transition to oral supplementation, resulting in correction of hypokalemia within 48 hours. Immunosuppressive therapy was initiated with intravenous methylprednisolone (1 g/day for three days), followed by oral prednisone (1 mg/kg/day). Hydroxychloroquine 200 mg twice daily was started for long-term disease control. Potassium citrate was administered for management of dRTA. Neurological improvement was rapid, with normalization of limb strength within 72 hours.

**Figure 2:**
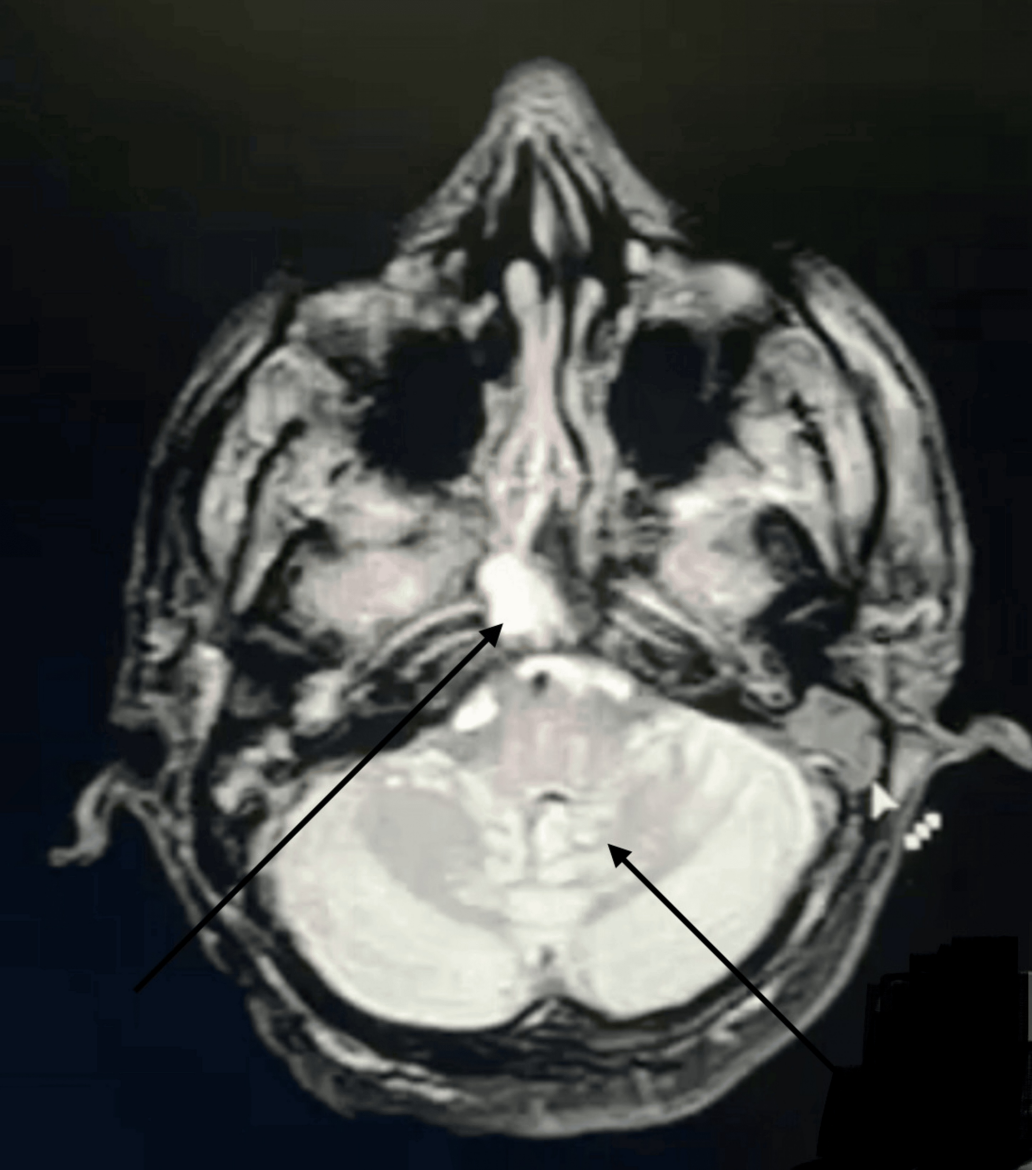
Follow-up axial T2-weighted MRI of the brain at eight weeks demonstrating residual signal changes in the central pons with reduced hyperintensity compared to the initial scan, consistent with resolving central pontine myelinolysis and residual gliosis. No gadolinium contrast was administered.

At six-month follow-up, the patient remained asymptomatic. Serum potassium was 4.1 mEq/L (reference range 3.5-5.0 mEq/L), and renal function was normal (serum creatinine 0.8 mg/dL). Neurological examination demonstrated full strength (MRC grade 5/5 in all limbs). Follow-up MRI brain showed stable residual pontine gliosis. She continued on hydroxychloroquine 200 mg twice daily and potassium citrate. Oral prednisolone was gradually tapered and discontinued. Follow-up MRI at eight weeks demonstrated residual pontine gliosis without evidence of active demyelination as shown in Figure 2.

## Discussion

SS can rarely present with severe electrolyte disturbances leading to neuromuscular complications. Hypokalemic paralysis represents an uncommon manifestation of SS, most often resulting from dRTA, which occurs in approximately 15-25% of patients due to autoimmune-mediated destruction of intercalated cells and dysfunction of H⁺-ATPase pumps in the collecting ducts [1-8]. The resulting hypokalemia arises from multiple mechanisms, including secondary hyperaldosteronism (as reflected by elevated renin levels in our patient), impaired H⁺/K⁺ exchange in dRTA, and bicarbonaturia-induced renal potassium wasting [9]. Although muscle weakness is common when serum potassium levels fall below 2.5 mEq/L, acute flaccid quadriparesis occurs in fewer than 5% of hypokalemic presentations [7].

Another notable aspect of this case is the occurrence of CPM in the absence of hyponatremia. Classically, CPM occurs as part of osmotic demyelination syndrome following rapid correction of chronic hyponatremia, particularly when serum sodium increases by more than 10-12 mEq/L within 24 hours [2,10]. In contrast, our patient maintained normonatremia throughout hospitalization and showed no evidence of significant osmotic shifts on serum or urine osmolality measurements.

Several mechanisms have been proposed to explain CPM occurring without hyponatremia, particularly in autoimmune diseases. Severe hypokalemia itself has been implicated as an independent risk factor for osmotic demyelination in normonatremic patients, as reported in multiple case series of CPM occurring without hyponatremia. This may occur through disruption of the Na⁺/K⁺-ATPase pump and endothelial dysfunction leading to astrocyte injury and demyelination [11]. Additionally, immune-mediated mechanisms may contribute in patients with SS, where circulating autoantibodies and inflammatory mediators could potentially target pontine oligodendrocytes. Reports of steroid-responsive cases further support an inflammatory component in such presentations [4-12]. Radiologically, the characteristic “trident sign” on MRI reflects selective vulnerability of central pontine oligodendrocytes to osmotic or inflammatory injury [5].

From a therapeutic perspective, CPM associated with autoimmune diseases such as SS may demonstrate a more favorable prognosis compared with classical osmotic demyelination syndrome. Neurological recovery has been reported in up to 80% of such cases, compared with approximately 50% recovery in traditional CPM [6-13]. Early recognition and prompt correction of electrolyte abnormalities remain essential. Furthermore, early immunosuppressive therapy with corticosteroids, as administered in our patient, may help mitigate immune-mediated demyelination and contribute to improved neurological outcomes [12]. This case also highlights the importance of screening for dRTA in patients with SS presenting with unexplained hypokalemia or neuromuscular weakness, as early identification and treatment may prevent severe complications such as hypokalemic paralysis and CPM [1].

Only a limited number of cases describing hypokalemic paralysis due to dRTA in SS have been reported in the literature [7]. However, the coexistence of CPM in the absence of hyponatremia in this setting remains exceedingly rare. Most reported cases of osmotic demyelination syndrome are associated with rapid correction of hyponatremia, whereas our patient maintained normonatremia throughout hospitalization. This case therefore highlights a rare neurological complication of SS and suggests that severe hypokalemia itself may act as an independent precipitating factor for osmotic demyelination.

## Conclusions

This case expands the spectrum of neurological manifestations of SS by demonstrating that severe hypokalemia resulting from dRTA can precipitate both acute hypokalemic paralysis and CPM, even in the absence of hyponatremia. The characteristic “trident sign” on MRI remains highly suggestive of CPM, even in atypical metabolic contexts. Clinicians should consider SS in middle-aged women presenting with unexplained electrolyte disturbances and CNS lesions, as early recognition and immunosuppressive therapy may improve neurological outcomes.

This case underlines the need to consider dRTA in patients with SS presenting with unexplained hypokalemia, as early recognition may help prevent serious complications; however, further studies are needed to better define the role of routine evaluation in this setting.

Long-term follow-up is important because approximately 30-50% of patients with primary SS develop systemic manifestations over time. Our patient remains asymptomatic on hydroxychloroquine and alkali therapy at six-month follow-up.

## References

[1] Ram R, Swarnalatha G, Dakshinamurty KV (2014). Renal tubular acidosis in Sjögren's syndrome: a case series. Am J Nephrol.

[2] Liu YC, Yang YK, Chen PS, Chang WH (2021). Central pontine myelinolysis in a normonatremic patient with depression. Clin Psychopharmacol Neurosci.

[3] Abdulla MC, Alungal J, Ahammed S, Narayan R (2017). Central pontine myelinolysis in Sjogren's syndrome with hypokalemia. Int J Rheum Dis.

[4] Shiboski CH, Shiboski SC, Seror R (2017). 2016 American College of Rheumatology/European League Against Rheumatism classification criteria for primary Sjögren's syndrome: a consensus and data-driven methodology involving three international patient cohorts. Arthritis Rheumatol.

[5] Beh SC (2017). Temporal evolution of the trident and piglet signs of osmotic demyelination syndrome. J Neurol Sci.

[6] Perzyńska-Mazan J, Maślińska M, Gasik R (2018). Neurological manifestations of primary Sjögren's syndrome. Reumatologia.

[7] Sarma A (2018). Hypokalemic paralysis due to primary Sjögren syndrome. Indian J Endocrinol Metab.

[8] Maripuri S, Grande JP, Osborn TG, Fervenza FC, Matteson EL, Donadio JV, Hogan MC (2009). Renal involvement in primary Sjögren's syndrome: a clinicopathologic study. Clin J Am Soc Nephrol.

[9] Walsh SB, Shirley DG, Wrong OM, Unwin RJ (2007). Urinary acidification assessed by simultaneous furosemide and fludrocortisone treatment: an alternative to ammonium chloride. Kidney Int.

[10] Verbalis JG, Goldsmith SR, Greenberg A, Korzelius C, Schrier RW, Sterns RH, Thompson CJ (2013). Diagnosis, evaluation, and treatment of hyponatremia: expert panel recommendations. Am J Med.

[11] Sonali S, Manali C, Rohidas B (2022). Osmotic demyelination syndrome as a manifestation of hypokalemia secondary to Sjogren’s syndrome with renal tubular acidosis. Indian J Neurol.

[12] Sterns RH, Hix JK, Silver SM (2013). Management of hyponatremia in the ICU. Chest.

[13] Brito-Zerón P, Baldini C, Bootsma H (2016). Sjögren syndrome. Nat Rev Dis Primers.

